# Psychological and Spiritual Well-Being Among Church of England Clergy and Laity three years After the COVID-19 Lockdowns

**DOI:** 10.1007/s10943-025-02412-5

**Published:** 2025-08-19

**Authors:** Andrew Village, Leslie J. Francis

**Affiliations:** 1 School of Humanities, York St John University, York, UK; 2 Centre for Research in Intellectual and Developmental Disabilities (CIDD), University of Warwick, Coventry, UK; 3 World Religions and Education Research Unit, Bishop Grosseteste University, Lincoln, UK

**Keywords:** Balanced affect, Clergy, COVID-19 lockdowns, Lay people, Psychological wellbeing, Spiritual wellbeing

## Abstract

The COVID-19 pandemic lockdowns caused declines in psychological wellbeing among both clergy and lay people in the Church of England. This study uses a convenience sample of 3,826 clergy and lay people from the *Church 2024* survey to examine perceived changes in psychological and spiritual wellbeing since the end of the 2021 lockdown. For most people negative affect either remained the same or decreased, and positive affect and spiritual wellbeing remained the same or increased, between 2021 and 2024. Analyses of scores for The Index of Balanced Affect Change (TIBACh) and the Spiritual Wellbeing Change Scale (SWCS) suggested that these improvements were not universal, and for some wellbeing may have changed little, or deteriorated between 2021 and 2024. Those that suffered most during the pandemic may have seen the greatest improvement after it ended, especially for younger people, stipendiary clergy, and men compared to women.

## Introduction

### Psychological Wellbeing and the COVID-19 Pandemic

The COVID-19 corona virus emerged in China in 2019 and spread rapidly over the next two years, causing a pandemic that had infected nearly 800 million people and caused over 7 million deaths by March 2025 (WHO, 2025). From early in 2020, concerns were expressed not only about the physical effects of the virus on those who caught it, but about the wider effects on mental health and wellbeing caused by the social isolation that resulted from attempts to prevent the infections spreading. Early surveys in the UK suggested that some groups, such as those with pre-existing poor mental health, were particularly prone to decreased levels of mental health in the first lockdown (O'Connor et al., 2021). A separate longitudinal survey at that time suggested that those most vulnerable in the general population to declining mental health during the first lockdown were adults under 25, women, and people living with young children (Pierce et al., 2020). Similar findings, of decreased mental health unevenly distributed across the general population, were reported in early pandemic surveys elsewhere in Europe (Fiorillo et al., 2020; Pieh et al., 2020) and in the USA (Holman et al., 2020; Jewell et al., 2020; Twenge et al., 2021).

As the pandemic continued into 2021 and beyond, researchers began to publish material that looked at mental health and wellbeing during subsequent lockdowns. The results comparing mental health and wellbeing early and later in the pandemic suggested different outcomes depending on which aspects of mental health were being examined and the period over which comparisons were made. For example, a panel study of adults in the UK from March to May 2020 showed increases in suicidal ideation increased over three waves, whereas anxiety decreased, and wellbeing increased (O'Connor et al., 2021). However, as the UK went into further lockdowns in 2021, mental health deteriorated and reached levels similar to those at the start of the first lockdown (Wetherall et al., 2022).

The World Health Organization declared an end to COVID-19 as a public health emergency on 5 May 2023. Although the virus continues to circulate, natural attenuation and vaccination have meant that deaths have decreased dramatically among general populations since then. A few studies are now emerging that compare mental health and wellbeing during the pandemic with this ‘post-pandemic’ period. [Some earlier studies used the term ‘post-pandemic’ to refer to surveys conducted in 2021, when the pandemic was still at its height, rather than after May 2023, when the disease was no longer as lethal and social distancing and lockdowns were no longer in force (see, for example: Bajoulvand et al., 2022; Jamshaid et al., 2023; Persson et al., 2021; Svob et al., 2023). The term ‘post-pandemic’ in this study refers to a period after May 2023.] A study in England of adults aged 50+ found that positive aspects of psychological wellbeing increased after the end of the pandemic and were higher than pre-pandemic levels; levels of depression decreased from pandemic levels, but remained higher than pre-pandemic levels (Zaninotto et al., 2025). This improvement of mental wellbeing for older adults after the pandemic may be in contrast to the ongoing effects on poor wellbeing among younger people (Kiviruusu et al., 2024). A cross-sectional study of the general population in the USA found marked declines in anxiety and depression between the end of 2023 and the start of 2024 (Arnett & Mitra, 2024). The study showed that the higher levels of anxiety and depression among younger than older people observed during the pandemic persisted into 2024, so although those in their 70s or older returned to levels similar to 2019, emerging adults (aged 18–29) still had levels higher than those recorded pre-pandemic. The emerging picture since the worst effects of the pandemic have receded is of improving psychological wellbeing for some groups, but not others.

### Religion and Wellbeing in the COVID-19 Pandemic

The psychological wellbeing of religious groups followed many of the same trends as non-religious groups. For example, the greater strain among younger adults, and those with children living at home, was apparent in a sample of 4,449 people from the Church of England in 2020 (Village & Francis, 2021d). There are, however, particular ways in which an event such as the COVID-19 pandemic might negatively affect religious groups such as committed members of Christian churches. The closure of churches led to the loss of familiar patterns of worship and face-to-face gatherings of church communities. Although online forms of worship quickly emerged, these were seen by some as inadequate replacement for lost previous rituals (Edelman et al., 2021; Village & Francis, 2024a). The loss of access to church buildings in some jurisdictions was particularly difficult for those in Catholic rather than Reformed traditions (Village & Francis, 2021a). Ministers of religion have particular sources of psychological strain even in normal times (Sielaff et al., 2021). During lockdowns they had rapidly to find new ways of facilitating worship and trying to maintain pastoral ministry when demands were high, and circumstances prevented effective forms of social contact. A number of studies indicated a greater toll on the psychological wellbeing of clergy compared with lay people during the pandemic (Village & Francis, 2021d). In addition, stipendiary clergy working in parishes in the Church of England seemed to fare worse than other clergy (Tweedie & Graveling, 2024; Village & Francis, 2021e). Stipendiary clergy in the Episcopal Church in the USA showed higher levels of negative affect during 2021 than other groups of clergy (Francis & Village, 2023).

Another aspect of how the pandemic may have influenced religious people in particular is in relation to their spiritual wellbeing. Times of crisis are known sometimes to increase religious sentiment among normally non-religious people, and religious people may use spiritual resources as part of their coping mechanisms (Gall & Guirguis-Younger, 2013). There was evidence of this during the pandemic (Counted et al., 2022), and indeed there is some evidence of positive spiritual renewal among religious groups (Kowalczyk et al., 2020). Those that held to the idea that God was in control of events through the pandemic or acting through the pandemic tended to show better psychological wellbeing (Beyerlein et al., 2021; Village & Francis, 2023b). Spiritual wellbeing may be correlated with psychological wellbeing: among clergy and lay people in the Church of England in 2021, self-perceived increase in spiritual wellbeing was positively correlated with increases in positive affect and negatively correlated with increases in negative affect (Village & Francis, 2023a).

These studies suggest that the pandemic may have affected religious groups in ways that were both common to general populations and specific to their particular religious beliefs, traditions, and roles. As the pandemic ended, we might expect to see changes in psychological and spiritual wellbeing among religious groups that mirror changes in the general population, but also that reflect the particular factors relevant for those who belong to religious communities. This study tests this idea by examining psychological and spiritual wellbeing as measured in surveys of the Church of England in 2020 and 2021 during the COVID-19 pandemic, and subsequently after the end of the pandemic in 2024.

### COVID-19 in the UK Since 2020

The initial response of the UK government to the arrival of COVID-19 was to issue a stay-at-home order on the 23 March 2020 (Brown et al., 2021). This reduced the rate of infections, and restrictions began to ease in June of that year. In this first lockdown, the Church of England issued guidance that went beyond the prohibition of public worship by banning clergy from entering churches (McGowan, 2020). As death rates declined in late spring and early summer, restrictions were gradually eased and the first lockdown ended. In the second half of 2020, the different jurisdictions in the UK diverged in their approaches to gatherings and wearing facemasks. In England, a tiered system of restrictions was introduced that was applied locally depending on the rates of infection. As schools and universities returned for the autumn term, there was another rise in cases, prompting a second national lockdown in England during November, though this time schools, universities, and a range of businesses remained open. The surge of cases that followed relaxations in lockdown over the Christmas period led to a third national lockdown that began in early January 2021. Restrictions were less stringent for public worship, which was permissible as long as there was social distancing and worshippers wore face masks. Restriction for this final lockdown began to ease in March and were all but gone by July 2023. The advent of mass testing and vaccination during 2021 meant that there was no repeat of the peaks in COVID-19 deaths seen in May 2020 or February 2021 in the winter of 2021–2022 or thereafter (ONS, 2023a). The wearing of facemasks in public was recommended after the end of the 2021 lockdown, but has gradually declined, and by 2023, there was little evidence of social restrictions related to the COVID-19 pandemic (ONS, 2023b).

### Pandemic Studies of Psychological Wellbeing in the Church of England

There have been two main studies of psychological wellbeing in the Church of England during the pandemic. The first was part of the *Living Ministry* programme, which began before the pandemic and which was aimed at identifying what factors help or hinder clergy in their ministry (Church of England, 2024). The panel survey started in 2017 and included waves in February–March 2019, March 2021, and March 2023 (Tweedie & Graveling, 2024). The survey included the Warwick Edinburgh Mental Wellbeing Scale (Tennant et al., 2007), which was completed by 339 clergy in both Waves 2 and 3 and by 358 clergy in both Wave 3 and Wave 4 (McFerran & Graveling, 2021; Tweedie & Graveling, 2024). The results suggested that whereas incumbent and assistant status clergy both showed declines in mental wellbeing scores from 2019 to 2021 going into the pandemic, the change coming out the pandemic was different in the two groups. Assistant clergy showed increased wellbeing, whereas incumbents showed a slight decline (Tweedie & Graveling, 2024, Figure 13). The *Living Ministry* survey also included assessments of financial wellbeing and relationship wellbeing, which both declined after 2021.

The other main study of wellbeing in the Church of England during the pandemic came from the *Coronavirus, Church and You* survey in 2020 (Village & Francis, 2020) and the *COVID-19 & Church-21* survey in 2021 (Village & Francis, 2021b, 2021c). Both these surveys were aimed at clergy and lay people and included measures of psychological wellbeing based on the balanced affect model (Bradburn, 1969). The Index of Balanced Affect Change (TIBACh) was developed in order to assess perceived changes in positive and negative affect since the pandemic began (Francis & Village, 2021), and the results reported in a series of publications exploring the factors that predicted levels of perceived change in wellbeing among clergy and lay people. Analysis of 4,449 clergy and laity from the 2020 survey showed that those who tended to fare worse were younger people, those with children under 13 living at home, and those living in inner cities (Village & Francis, 2021d). These trends tended to reflect those seen among the wider general population at the time. Psychological dispositions also played a role, with better wellbeing among those who preferred feeling over thinking in their psychological type judging process and worse wellbeing among those with a general tendency towards neuroticism. In addition, there were differences related to aspects of religion, with Anglo-Catholics faring worse than others, Evangelicals faring better, and clergy faring worse than lay people. These results were also evident in the 2021 survey (Village & Francis, 2022b). Comparison of levels of perceived changes in positive and negative affect in the 2020 and 2021 surveys suggested that psychological wellbeing continued to decline as the pandemic persisted (Village & Francis, 2022a). As yet there has been no comparable study to test if psychological wellbeing, measured in terms of balanced affect, has changed since 2021.

### Pandemic Studies of Spiritual Wellbeing in the Church of England

Four items in the *Coronavirus, Church and You* survey were used to assess perceived changes in spiritual wellbeing since the onset of the pandemic: prayerfulness, closeness to God, closeness to the church, and personal faith, which were used as items in the Lewis Index of Spiritual Awakening (Francis et al., 2021b). Scores for this index among 1,050 Church of England clergy suggested more had experienced spiritual awakening than spiritual decline. For example, 50% reported being more prayerful since the start of the pandemic, compared to only 14% reporting being less prayerful. Similarly, 42% reported feeling closer to God compared to only 7% feeling further from God. Similar findings were apparent in a sample of 3,673 lay people from the same survey (Francis et al., 2022). These results suggested that spiritual and psychological wellbeing were not necessarily going in the same direction during the first lockdown.

The *COVID-19 & Church-21* survey used a different measure of perceived changes in spiritual wellbeing, the Spiritual Wellbeing Change Scale (SWCS) based on perceived changes in the frequencies of personal prayer and Bible reading, trust in God, the quality of spiritual life, and spiritual health (Village & Francis, 2023a). Analyses of a sample of 1,878 clergy and lay people from the Church of England suggested increases in spiritual wellbeing outweighed declines, with 48% reporting increased personal prayer compared to 17% reporting decline, and 42% reporting increased trust in God compared to 6% reporting decline. Scores on the SWCS were negatively correlated with negative affect and positively correlated with positive affect as measured by the TIBACh; however, the SWCS had independent effects on self-reported changes in both mental and physical health after controlling for psychological wellbeing, suggesting that it may have been an independent aspect of overall wellbeing during the pandemic in the Church of England.

### Objectives

Given the above, it would be useful to know how levels of psychological and spiritual wellbeing have changed among clergy and laity in the Church of England since the end of the 2021 lockdowns. The aim of this study was twofold:

First, to see if levels of psychological wellbeing (as measured by balanced affect) had changed among clergy and lay people in the Church of England since the 2020 and 2021 national lockdowns during the COVID-19 pandemic.

Second to examine in more detail changes in psychological and spiritual wellbeing since 2021. The particular aim here was to see if any recovery in wellbeing was more evident is some groups within the Church of England than in others.

## Method

### Procedure

The procedures and sample profiles for the 2020, 2021, and 2024 surveys are described in detail elsewhere (Village, 2025; Village & Francis, 2022a) and will not be repeated here. All three surveys were online and used the same methods to recruit participants based on a combination of repeated requests in national church newspapers and direct requests to dioceses. The online *Church 2024* survey ran from March to November 2024 using the Qualtrics platform. It was intended primarily to measure a wide range of attitudes and opinions as a follow-up from two previous *Church Times* surveys in 2001 and 2013 (Francis et al., 2005; Village, 2018). Included in the survey were some items used in the 2020 and 2021 COVID-19 surveys, which were intended to allow measurement of changes in wellbeing between the three surveys, and particularly between 2021 and 2024. The *Church 2024* survey was promoted in the Church of England and through Roman Catholic networks in the UK and the Republic of Ireland. Of the 5,141 total responses to the survey, 4,395 (85.5%) were people living in England, 171 (3.3%) elsewhere in the UK and Northern Ireland, 481 (9.4%) in the Republic of Ireland, and 95 (1.8%) elsewhere. In terms of religious affiliation, 4,044 (78.7%) were Anglicans, of which 3,826 (94.6%) were from the Church of England. Although this was a convenience sample, evidence suggests that it may have been a reasonable representation of lay people in the Church of England. For clergy, women may have been slightly over-represented, especially those with extra-parochial responsibilities (for details, see Village, 2025).

### Participant Profile

For compatibility with previous reports of psychological wellbeing in 2020 and 2021, a subsample of 1,760 was used which comprised stipendiary parochial clergy and non-ministering lay people (Table 1). This enabled direct comparison of psychological wellbeing with previous published results. Items for comparing spiritual wellbeing were present in the 2021 and 2024 surveys only.

**Table 1:** Profile of non-ministering laity and stipendiary parochial clergy in the three surveys

	NML	SPC	All	NML	SPC	All	NML	SPC	All
*N* =	2815	792	3607	1027	401	1428	1093	667	1760
	%	%	%	%	%	%	%	%	%
*Sex*									
Female	66	47	62	61	42	56	56	40	50
Male	34	53	38	39	58	45	44	60	50
*Age*									
20s	4	2	4	2	1	2	8	1	5
30s	7	14	8	4	9	5	10	13	11
40s	12	25	15	9	20	12	14	22	17
50s	19	36	23	16	36	22	18	36	25
60s	27	24	26	32	33	32	26	23	25
70s	26	0	20	32	1	23	21	4	14
80s+	5	0	4	6	0	4	5	1	4
*Tradition*									
Anglo-Catholic	27	35	29	27	29	28	27	31	28
Broad Church	54	46	52	56	45	53	52	43	50
Evangelical	19	19	19	17	26	19	21	27	23
*Location*									
Rural	34	35	34	37	36	36	34	30	32
Town/suburb	57	53	56	57	53	56	56	58	57
Inner city	9	12	10	7	11	8	10	12	11

NML, non-ministering laity; SPC, stipendiary parochial clergy

For the more detailed analyses comparing psychological and spiritual wellbeing changes in 2021 and 2024, all categories of clergy and lay people were included in both years, resulting in a total sample of 5,092 (1,865 in 2021 and 3,227 in 2024, Table 2).

**Table 2:** Profile of samples used to compare 2021 and 2024 surveys

	2021	2024			
*N* =	1865	3227			
	%	%	χ^2^		*P*
*Sex*					
Female	55	52			
Male	45	48	5.8	1	*
*Age*					
20s	1	4			
30s	4	8			
40s	10	13			
50s	20	21			
60s	35	26			
70s	26	22			
80s+	5	6	107.7	6	***
*Household status*					
Live with others	78	81			
Live alone	22	19	6.1	1	*
No children	86	85			
Children	14	15	1.3	1	NS
No teenagers	91	88			
Teenagers	9	12	14.8	1	***
*Location*					
Rural	37	33			
Town	32	33			
Suburban	24	24			
Inner city	8	11	16.7	3	***
*Church status*					
Stipendiary parochial	20	21			
Stipendiary extra-parochial	2	2			
Active SSM or retired	14	13			
Lay minister	14	17			
Not ministering	50	47	13.7	4	**
*Tradition*					
Anglo-Catholic	29	28			
Broad Church	51	44			
Evangelical	20	27	35.9	2	***

Differences between 2021 and 2024 were tested with contingency tables. *p < .05; **p < .01; ***p < .001, NS not significant

### Instruments

#### Psychological Wellbeing

The Index of Balanced Affect Change (TIBACh) comprised ten items, five related to positive affect, PA, and five to negative affect, NA (Francis & Village, 2021). Respondents were asked to indicate if affect such as happiness, stress, or anxiety had increased, stayed the same, or decreased. In 2020, there was a three-point response scale, but this was changed to a five-point scale in 2021 and 2024. In 2020 and 2021, items were introduced by the rubric ‘How would you rate how you are now compared with before the pandemic started?’. In 2024, the rubric was ‘How would you rate how you are now compared with how you were during the pandemic lockdowns?’ For analyses comparing all three surveys, the two responses at either end of the five-point scale 2021 and 2024 were collapsed to produce a three-point scale. For analyses comparing the 2021 and 2024 surveys, the five-point scales were retained. Alpha reliabilities for the five-point scale for the combined 2021 and 2024 samples were PA: .84 and NA: .86.

#### Spiritual Wellbeing

The Spiritual Wellbeing Change Scale (SWCS) was developed from the 2021 survey and consisted of five items with a five-point response scale (Village & Francis, 2023a). Items referred to changes in the frequency of personal prayer, frequency of bible reading, trust in God, quality of spiritual life, and overall spiritual health. For 2021, this was asked since the pandemic began; for 2024 this was asked since the pandemic ended. Alpha reliability for the combined 2021 and 2024 sample used in this analysis was .84.

#### Predictor Variables

For the comparison of the 2021 and 2024 surveys, variables previously shown to predict psychological affect and/or spiritual wellbeing were included in multiple regression models. Some items used in 2021, such as details about COVID-19 infection, were not included in 2024 as they were no longer relevant. Predictor variables present in both surveys were sex (1 = female; 0 = male), age (by decade 2 = 20s to 8 = 80s+), household status (1 = living alone; 0 = lives with others), children (under 13) living at home (1 = yes; 0 = no) and church tradition (1 = Broad Church, 2 = Anglo-Catholic, 3 = Evangelical).

Both survey datasets included a ministry status variable that categorized clergy into stipendiary parochial, stipendiary extra-parochial, or self-supporting/retired with permission to officiate, and laity into lay ministers and non-ministering laity (which included a few non-ministering clergy). Initial analyses showed that most categories had similar levels of the three dependent variables, apart from stipendiary parochial clergy, so a dummy variable was used to identify this group in the regressions comparing the 2021 and 2024 surveys (1 = stipendiary parochial clergy; 0 = other clergy and laity).

Psychological variables were assessed using the Francis Psychological Type and Emotional Temperament Scales revised version, FPTETS-R (Village & Francis, 2023c, 2023d). The 2024 survey used the revised shortened version (Village & Francis, 2024c), so the corresponding items from the 2021 survey (which had the full version) were used to calculate scores for extraversion, intuition, feeling, judging, and emotional volatility. Alpha reliabilities for these six-item scales in the combined 2021 and 2024 samples were: extraversion: .81, intuition: .68, feeling: .78, judging: .78, and emotional volatility: .82.

### Analysis

The first stage of analysis was to compare the response frequencies for the ten psychological affect items common to all three surveys. Changes in PA and NA from 2020 to 2021 have been reported elsewhere, and data are repeated here to demonstrate how perceived changes in psychological wellbeing since the pandemic compare with perceived changes during the pandemic. The statistical significance of changes in item responses from 2021 to 2024 were tested using 3 × 2 contingency tables with two degrees of freedom. Changes in responses to items in the SWCS between 2021 and 2024 were treated in the same way.

The second stage of analysis was to explore in more detail the changes in PA, NA, and SWCS from 2021 to 2024. The balanced affect scales based on five-point response scales were used for this analysis. Multiple regression employed the generalized linear model procedure of SPSS 29 (IBM_SPSS, 2023) to identify the significant independent effects predicting each of the three measures of wellbeing, and to test for changes between the two surveys. Age was treated as a continuous variable and centred on five (= 50s); psychological variables were mean centred. The model included interaction effects for sex, age, location (rural and inner city), household status (living alone and children in household), church tradition, and ordination status (stipendiary parochial clergy) against survey number. The interactions tested whether or not changes in wellbeing between surveys were of equal magnitudes across groups, or whether some groups showed relatively more change than others. Significant interactions were displayed graphically using parameter estimates from the linear model.

## Results

### Changes in Individual Affect Items

For both lay people (Table 3) and clergy (Table 4), the trend was for reductions in positive affect and increases in negative affect between 2020 and 2021 (as reported in Village & Francis, 2022a), followed by increases in positive affect and decreases in negative affect from 2021 to 2024.

**Table 3:** Changes in affect item responses between surveys for non-ministering laity

	Survey									2021
	2020			2021			2024			versus
*N* =	2815			1027			1093			2024
	Less	Same	More	Less	Same	More	Less	Same	More	*χ*^2^
	%	%	%	%	%	%	%	%	%	
(a) *Positive affect*										
Excited	35	59	6	57	37	6	15	54	30	466.4***
Happy	25	60	15	39	50	11	8	56	36	381.7***
Confident	13	69	18	27	56	17	12	55	33	117.9***
Hopeful	21	55	25	23	43	34	11	47	42	58.6***
Thankful	4	39	56	6	36	58	3	45	52	18.5***
(b) *Negative affect*										
Frustrated	10	49	41	9	31	60	21	54	25	267.4***
Anxious	18	45	37	10	41	49	35	47	18	318.9***
Exhausted	23	48	29	15	45	40	19	51	30	26.5***
Stressed	23	45	33	17	41	42	28	50	22	100.4***
Fatigued	18	43	40	14	40	46	19	49	32	42.4***

Differences between 2021 and 2024 were tested with contingency tables with 2 df. ***p < .001

**Table 4:** Changes in affect item responses between surveys for stipendiary parochial clergy

	Survey									2021
	2020			2021			2024			versus
*N* =	792			401			667			2024
	Less	Same	More	Less	Same	More	Less	Same	More	*χ*^2^
	%	%	%	%	%	%	%	%	%	
(a) *Positive affect*										
Excited	34	50	16	56	31	14	20	35	45	171.5***
Happy	26	56	17	41	50	9	16	44	40	149.1***
Confident	11	63	26	27	49	24	13	43	44	56.2***
Hopeful	16	50	34	22	38	40	13	40	47	14.5***
Thankful	4	38	58	7	41	52	6	42	52	1.0
(b) *Negative affect*										
Frustrated	11	38	51	7	26	67	25	40	35	118.7***
Anxious	19	40	41	10	33	57	37	42	21	168.6***
Exhausted	16	25	58	10	17	73	25	33	42	101.9***
Stressed	20	35	45	13	27	60	25	40	35	76.9***
Fatigued	13	22	65	6	12	81	19	33	47	132.1***

Differences between 2021 and 2024 were tested with contingency tables with 2 df. ***p < .001

For positive affect among lay people, 57% reported in 2021 feeling less excited since the pandemic began and only 6% felt more excited, whereas in 2024 only 15% felt less excited since the pandemic ended and 30% felt more excited (Table 3a). Similar trends were seen for happiness, confidence, and hopefulness. The exception was thankfulness, which had been high throughout the pandemic and seemed to remain high post-pandemic. For negative affect among lay people, 60% reported in 2021 feeling more frustrated, and 9% less frustrated, since the pandemic began, whereas in 2024 25% reported feeling more frustrated, and 21% less frustrated, since the pandemic ended (Table 3b). Similar trends were apparent in other negative affect items, though it was notable that almost a third of the sample reported feeling more exhausted and more fatigued since the pandemic ended, which was lower than in 2021, but still relatively high.

For positive affect among stipendiary parochial clergy, 56% reported in 2021 feeling less excited since the pandemic began and 14% felt more excited, whereas in 2024 20% felt less excited since the pandemic ended and 45% felt more excited (Table 4a). Similar trends were seen for happiness, confidence, and hopefulness. The exception was again thankfulness, which had been high throughout the pandemic, and there was no change since then. For negative affect among clergy, 67% reported in 2021 feeling more frustrated, and 7% less frustrated, since the pandemic began, whereas in 2024 35% reported feeling more frustrated, and 25% less frustrated, since the pandemic ended (Table 4b). Similar trends were apparent in other negative affect items, though as with lay people, a sizable minority reported feeling more fatigued (47%) and exhausted (42%) since the pandemic ended. Clearly, for some parish-based clergy the demands of ministry may have increased rather than decreased after the pandemic.

Overall, these results showed a consistent pattern: between the first and second years of the pandemic there was a growth in negative affect and a decline in positive affect, especially among stipendiary parochial clergy when compared with non-ministering lay people. From the end of lockdowns in 2021 and 2024, most people reported having similar or increased levels of positive affect and similar or decreased levels of negative affect. This indicates some improvement in psychological wellbeing, but not a complete reversal to what might have been pre-pandemic levels. There were still sizeable minorities who felt increases in negative affect, and smaller minorities who perceived lower levels of positive affect, since the end of the pandemic lockdowns.

### Changes in Individual Spiritual Wellbeing Items 2021 to 2024

Changes in the frequency of response for the five items in the SWCS suggested most people felt there was little change since the end of the pandemic (Table 5). For laity, 58% recorded ‘same’ for personal prayer and 62% for bible reading (Table 5a). The quality of spiritual life and spiritual health both showed fewer people recording a decrease and more recording the same or increase in 2024 compared to 2021. Stipendiary parochial clergy recorded similar trends (Table 5b). For both groups, the least changed item was ‘My trust in God’.

**Table 5:** Changes in Spiritual Wellbeing Change Scale item responses from 2021 to 2024

	Survey						
	2021			2024			
	Less	Same	More	Less	Same	More	
	%	%	%	%	%	%	χ^2^
(a) *Non-ministering laity*							
*N* =		939			1508		
Frequency of personal prayer	15	38	47	13	58	30	92.0***
Frequency of bible reading	16	57	27	16	62	22	8.8*
Quality of my spiritual life	26	37	37	15	47	39	50.9***
My trust in God	8	55	37	7	54	39	1.7
Spiritual health	21	41	38	12	44	44	40.9***
(b) *Stipendiary parochial clergy*							
*N* =		365			675		
Frequency of personal prayer	23	35	42	18	52	30	26.5***
Frequency of bible reading	20	48	32	17	59	24	10.1**
Quality of my spiritual life	35	30	35	19	44	38	36.3***
My trust in God	3	47	50	5	46	49	1.7
Spiritual health	26	41	33	13	41	46	32.7***

### Changes in Psychological and Spiritual Wellbeing from 2021 to 2024

The 2021 and 2024 surveys used the same items and response scales for affect and spiritual wellbeing, so these could be investigated more thoroughly. After controlling for the different profiles of the two surveys, the multiple regression confirmed the decline in NA and increase in PA between 2021 and 2024 (Table 6). In addition, there was a smaller but significant increase in the overall SWCS score.

**Table 6:** Multiple regression of negative affect, positive affect, and spiritual wellbeing change scales

Predictor	Category	Parameter estimates (SE)		
		Negative affect	Positive affect	Spiritual wellbeing
Survey (2021)	2024	− 2.85 (0.24)***	1.99 (0.22)***	0.74 (0.25)**
Sex (male)	Female	− 0.06 (0.17)	− 0.11 (0.15)	0.37 (0.17)*
Age		− 0.48 (0.07)***	0.20 (0.07)**	0.23 (0.07)**
Extraversion		− 0.03 (0.02)	0.10 (0.02)***	0.06 (0.03)*
Intuition		− 0.09 (0.03)**	0.12 (0.03)***	0.16 (0.03)***
Feeling		− 0.01 (0.03)	0.09 (0.02)***	0.15 (0.03)***
Judging		− 0.12 (0.03)***	0.11 (0.03)***	0.12 (0.03)***
Emotional volatility		0.51 (0.03)***	− 0.36 (0.02)***	− 0.20 (0.03)***
Living status (with others)	Live alone	− 0.31 (0.20)	0.34 (0.18)	0.53 (0.20)**
Children (none)	Yes	0.17 (0.24)	− 0.17 (0.22)	0.15 (0.25)
Location (not rural)	Rural	− 0.21 (0.17)	− 0.13 (0.15)	− 0.23 (0.17)
Location (not inner city)	Inner city	− 0.26 (0.31)	− 0.03 (0.28)	0.10 (0.32)
Ordination (not Stipar)	Stipendiary parochial	1.30 (0.21)***	− 0.03 (0.19)	− 0.24 (0.22)
Church Tradition (BC)	Evangelical	− 0.53 (0.21)*	0.62 (0.19)**	0.58 (0.22)**
	Anglo-Catholic	0.13 (0.18)	− 0.17 (0.17)	− 0.25 (0.19)
Sex * survey	Female * 2024	0.14 (0.20)	0.42 (0.19)*	− 0.76 (0.21)***
Age * survey	Age * 2024	0.37 (0.08)***	− 0.42 (0.08)***	− 0.42 (0.09)***
Rural * survey	Rural * 2024	0.49 (0.21)*	0.15 (0.20)	0.44 (0.22)*
Inner city * Survey	Inner city * 2024	0.12 (0.37)	0.29 (0.34)	0.02 (0.38)
Church tradition * survey	Evangelical * 2024	0.27 (0.26)	− 0.22 (0.23)	0.24 (0.26)
	Anglo-Catholic * 2024	− 0.40 (0.23)	0.35 (0.22)	0.43 (0.24)
Live alone * survey	Live alone * 2024	0.24 (0.25)	− 0.53 (0.23)*	− 0.47 (0.26)
Children * survey	Children * 2024	− 0.09 (0.30)	0.29 (0.28)	− 0.36 (0.31)
Ordained * survey	Stipar * 2024	− 0.66 (0.26)*	0.12 (0.24)	− 0.37 (0.27)

Reference categories for categorical predictors are in parentheses. Stipar = stipendiary parochial clergy, BC = Broad Church

*p < .05; **p < .01; ***p < .001, otherwise not significant

**Fig. 1:**
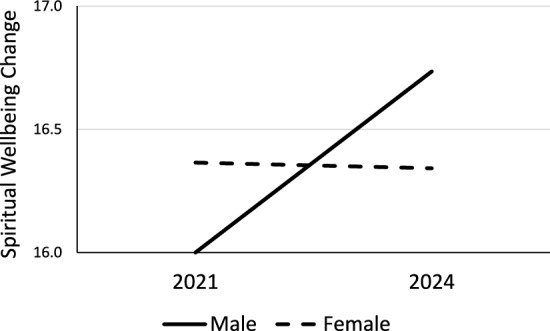
Spiritual Wellbeing Change Scale scores for men and women in 2021 and 2024

In terms of predictor variables, the following trends were apparent across the combined sample from 2021 and 2024:

There was no significant difference in negative or positive affect scores between men and women, but women showed slightly higher average scores for spiritual wellbeing change than men. The interactions suggested that men may have improved their spiritual wellbeing slightly more, on average, than did women after the pandemic ended (Fig. 1).

**Fig. 2:**
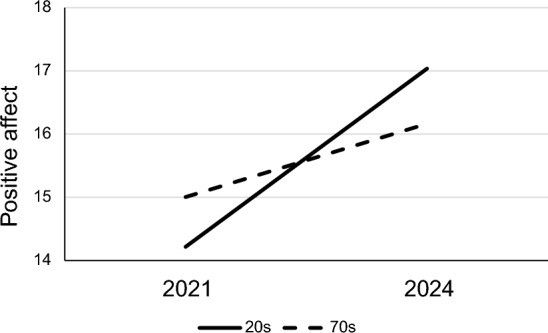
Positive affect scores by age in 2021 and 2024

The age effects showed that, across both surveys, younger people tended to have higher NA and lower PA or SWCS than older people. This was in line with findings during the pandemic, but the interactions suggested that the increases in psychological and spiritual wellbeing between surveys were greater among young people than among old people. For example, both those in their 20s and those in their 70s showed increases in PA between surveys, but the more rapid change among the younger group meant that they went from having lower PA than older people in the pandemic to having higher PA after it (Fig. 2).

The effects of personality variables were similar to those found during the pandemic. In particular, the tendency was for PA and SWCS scores to be more closely related to psychological disposition than were NA scores. For the combined 2021 and 2024 surveys, PA and SWCS scores were higher among extraverts than introverts, among intuitive than among sensing types, among feeling types than among thinking types, and among judging types than among perceiving types. They were negatively correlated with emotional volatility.

**Fig. 3:**
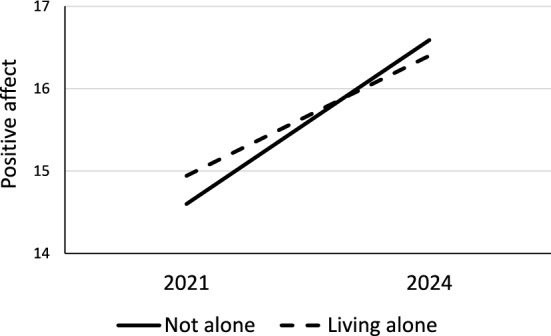
Positive affect scores by household status in 2021 and 2024

Living with others in the household had no overall relationship to wellbeing scores, but there was a small but statistically significant interaction effect on PA by survey, which suggested that those living alone had slightly better scores in 2021 than those living with others, whereas their scores were similar, or slightly lower than those living with others in 2024 (Fig. 3).

**Fig. 4:**
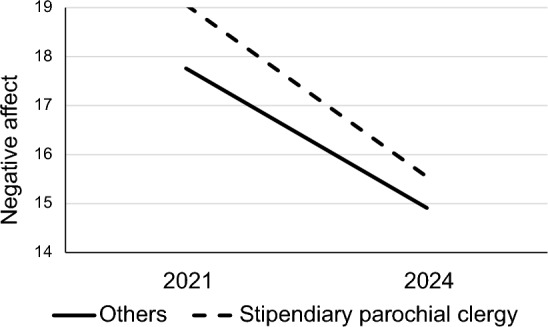
Negative affect scores by ordination status in 2021 and 2024

The greater NA of stipendiary parochial clergy compared with other clergy or laity during the pandemic was noted in the 2021 survey (Village & Francis, 2022b) and was repeated in the combined 2021 and 2024 data. However, there was a slight, but significant, trend for such clergy to show a greater rate of decline in NA compared with others, though this was not enough to reverse or remove the trend seen during the pandemic (Fig. 4). In terms of church tradition, Evangelicals tended to have lower NA and higher PA and SWCS scores than either Broad Church or Anglo-Catholic, and this was consistent between the two surveys.

Overall, the multiple regression results confirm the tendency for reduced negative affect and increased positive affect and spiritual wellbeing between 2021 and 2024. Some trends persisted across both surveys, such as the better psychological and spiritual wellbeing of older than younger people, the tendency for psychological variables to have greater correlations with positive affect or spiritual wellbeing rather than negative affect, and the better wellbeing of Evangelicals compared with others. What was also revealed was the that some groups that were most seriously affected by the pandemic, such as men, younger people, and stipendiary clergy, showed greater rates of improvement after the pandemic ended.

## Discussion

The main aim of the study was to investigate how psychological wellbeing (as measured by changes in positive and negative affect) and spiritual wellbeing have changed among clergy and laity in the Church of England since the end of the COVID-19 lockdowns in 2021. The results suggest that, as might be expected, more people perceived increases in positive affect and decreases in negative affect than was apparent during the second year of pandemic lockdowns in 2021. In that year, negative affect had increased, and positive affect decreased for both clergy and lay people, suggesting that there was little sign of people getting used to locked-downed society, even though access to churches was better in 2021 than in 2020. Since the end of the pandemic life has returned to something akin to a pre-pandemic state (at least in terms of high mortality and social restrictions), and this seems to have improved psychological wellbeing for some in the sample.

Although there were overall improvements in psychological wellbeing by 2024, these seemed to be relatively modest for both clergy and lay people because well over half reported the same or less levels of positive affect or the same or more negative affect compared with during the pandemic. These are subjective assessments, but they may reflect a lingering perception that life has not simply reverted to what it was before the pandemic. Since the pandemic, global, UK secular, and church life have had suffered events that may have reduced a sense of wellbeing, so these may have left people in the Church of England with lower subjective wellbeing than they might otherwise have had. Notably, exhaustion and fatigued, which reached high levels during the pandemic, seemed to have declined only marginally, especially among stipendiary clergy. In 2021, 81% felt more fatigued and 73% more exhausted since the pandemic started. In 2024, 47% felt more fatigued and 42% more exhausted since the pandemic ended (Table 4b). This is in line with other surveys of Church of England incumbent status clergy (Tweedie & Graveling, 2024) and suggests that the Church needs to address this problem with some urgency. The *Church 2024* survey included items measuring perceptions of the fragility churches (Francis et al., 2021a), and these suggested clergy especially are still concerned about the viability of their churches (unpublished). Financial pressures have intensified since the pandemic (Hughes & Woolcock, 2024), and this may be an ongoing source of stress in local churches.

During the pandemic, it was noticeable that certain groups in the Church tended to report lower psychological wellbeing than did others. In particular, it was young people, especially those with children at home, who felt the effects of lockdowns more than older, retired people. Stipendiary parish clergy also showed lower wellbeing (especially greater increase in negative affect) than either lay people or clergy in other roles, reflecting the difficulties of maintaining ministry when society and churches were locked down. The greater reduction in negative affect among this group of clergy suggests they may be recovering from the particular pressures of the pandemic, though they still had higher levels of negative affect change than any other group in the Church of England.

The 2020 COVID-19 survey suggested that, for some churchgoers, lockdown life may have actually improved their spiritual wellbeing (Francis et al., 2021b; Francis et al., 2022). Equivalent findings have been reported in some other studies (Büssing et al., 2021; Hyde & Joseph, 2022; Joseph & Hyde, 2022; Ruan et al., 2023) and may reflect the way that religious faith and practice can increase during times of adversity. The 2021 survey responses to the SWCS suggested that some 30–50% of laity and stipendiary parochial clergy reported increases since the pandemic began, and a large majority reported the same or increase for the same items in 2024. This was not a longitudinal study, and these responses are subjective perceptions of changes in personal spirituality, but they suggest that any spiritual awakening during the pandemic may have been maintained or increased thereafter. As reported for the 2021 survey (Village & Francis, 2023a, 2024b), spiritual wellbeing was associated with personal and psychological factors. Changes from 2021 to 2024 suggested that men, who reported poorer spiritual wellbeing than women during the pandemic, may have seen more increase in spiritual wellbeing than women when the lockdowns ended. The same trend was apparent when comparing younger with older people.

### Conclusions

This comparison of self-perceived changes in psychological and spiritual wellbeing among members of the Church of England from the 2021 COVID-19 pandemic lockdown to the post-pandemic year of 2024 indicated several important findings:

First, most people reported that negative affect had either remained the same or decreased, and positive affect and spiritual wellbeing had remained the same or increased, between 2021 and 2024. Scores for The Index of Balanced Affect Change (TIBACh) and Spiritual Wellbeing Change Scale (SWCS) suggested that there had been some overall improvement in both psychological and spiritual wellbeing in the 3 years after the worst of the pandemic.

Second, the scale of the changes suggested that these improvements were not universal, and for some wellbeing may have changed little, or deteriorated since 2021. The data collected here do not show why this might be, but anecdotal evidence from some churchgoers and clergy suggests that this might partly be because of the long-term effects of on the pandemic on church life, as well as ongoing issues in wider society. More detailed work is needed to understand who has or has not seen improvements in wellbeing and why this might be.

Third, changes from 2021 to 2024 were not always uniform across different groups, and those that suffered most during the pandemic may have seen the greatest improvement, as with all measures between young and old, negative affect changes for stipendiary clergy, and spiritual wellbeing changes for men compared to women. These may point to the particular effect of the lockdowns that made heavier demands on some than on others. When the conditions alleviated, these groups gained the most benefit. Meanwhile, the underlying predictors of wellbeing, especially those related to psychological dispositions, remained fairly consistent, so factors that predicted better or worse wellbeing changes in 2021 were also likely to predict them in 2024.

### Limitations of the Study

The study was based on surveys that gathered convenience samples. Although the available evidence suggests both clergy and lay samples were reasonably representative of the Church of England, it is difficult to determine the exact profile of the Church as whole, so we could not tell how generalizable the results are to the whole membership. The measures of psychological and spiritual wellbeing were based on subjective perceptions of change over time, rather than absolute measures at a point in time. Some individuals may have been better a gauging change than others, so the results may not have been the same if other measures had been used. Clearly, the best data would be longitudinal panel data that employ instruments based on the same theoretical constructs of balanced affect and spiritual wellbeing within a Christian context. To that end, churches should consider measuring levels of psychological and spiritual wellbeing to give base levels that can be used to explore the effects of trauma in the future.
